# Supplementary material for: Cytotoxic activity of Ganoderma weberianum-sichuanese isolated from the Lower Volta River Basin of Ghana against human prostate carcinoma (PC-3), leukemic T cell (Jurkat), and plasmacytoid dendritic cell (pDC)-derived acute leukemia (PMDC05) cell lines
Source: PLoS One. 2025 Jul 11;20(7):e0327087. doi: 10.1371/journal.pone.0327087 (PMC12250544; doi:10.1371/journal.pone.0327087)
Supplement: S1 Table — (PDF) [file pone.0327087.s001.pdf]

Multiple Comparison IC_{50} of Jurkat

Tukey HSD

(I) Fractions	(J) Fractions	Mean Difference (I-J)	Std. Error	Sig.	95% Confidence Interval	
					Lower Bound	Upper Bound
CURCUMIN	GL-C1	-57.45133*	6.17434	.000	-79.0854	-35.8173
	GL-C2	-14.74133	6.17434	.346	-36.3754	6.8927
	GL-C3	-46.46933*	6.17434	.000	-68.1034	-24.8353
	GL-C4	-97.64700*	6.17434	.000	-119.2810	-76.0130
	GL-C5	-97.64700*	6.17434	.000	-119.2810	-76.0130
	GL-C6	-97.64700*	6.17434	.000	-119.2810	-76.0130
	GL-C7	-97.64700*	6.17434	.000	-119.2810	-76.0130
	GL-C0	-997.64700*	6.17434	.000	-1019.2810	-976.0130
GL-C1	CURCUMIN	57.45133*	6.17434	.000	35.8173	79.0854
	GL-C2	42.71000*	6.17434	.000	21.0760	64.3440
	GL-C3	10.98200	6.17434	.694	-10.6520	32.6160
	GL-C4	-40.19567*	6.17434	.000	-61.8297	-18.5616
	GL-C5	-40.19567*	6.17434	.000	-61.8297	-18.5616
	GL-C6	-40.19567*	6.17434	.000	-61.8297	-18.5616
	GL-C7	-40.19567*	6.17434	.000	-61.8297	-18.5616
	GL-C0	-940.19567*	6.17434	.000	-961.8297	-918.5616
GL-C2	CURCUMIN	14.74133	6.17434	.346	-6.8927	36.3754
	GL-C1	-42.71000*	6.17434	.000	-64.3440	-21.0760
	GL-C3	-31.72800*	6.17434	.002	-53.3620	-10.0940
	GL-C4	-82.90567*	6.17434	.000	-104.5397	-61.2716
	GL-C5	-82.90567*	6.17434	.000	-104.5397	-61.2716
	GL-C6	-82.90567*	6.17434	.000	-104.5397	-61.2716
	GL-C7	-82.90567*	6.17434	.000	-104.5397	-61.2716
	GL-C0	-982.90567*	6.17434	.000	-1004.5397	-961.2716
GL-C3	CURCUMIN	46.46933*	6.17434	.000	24.8353	68.1034
	GL-C1	-10.98200	6.17434	.694	-32.6160	10.6520
	GL-C2	31.72800*	6.17434	.002	10.0940	53.3620
	GL-C4	-51.17767*	6.17434	.000	-72.8117	-29.5436
	GL-C5	-51.17767*	6.17434	.000	-72.8117	-29.5436
	GL-C6	-51.17767*	6.17434	.000	-72.8117	-29.5436
	GL-C7	-51.17767*	6.17434	.000	-72.8117	-29.5436
	GL-C0	-951.17767*	6.17434	.000	-972.8117	-929.5436
GL-C4	CURCUMIN	97.64700*	6.17434	.000	76.0130	119.2810
	GL-C1	40.19567*	6.17434	.000	18.5616	61.8297
	GL-C2	82.90567*	6.17434	.000	61.2716	104.5397
	GL-C3	51.17767*	6.17434	.000	29.5436	72.8117
	GL-C5	.00000	6.17434	1.000	-21.6340	21.6340
	GL-C6	.00000	6.17434	1.000	-21.6340	21.6340

	GL-C7	.00000	6.17434	1.000	-21.6340	21.6340
	GL-C0	-900.00000*	6.17434	.000	-921.6340	-878.3660
GL-C5	CURCUMIN	97.64700*	6.17434	.000	76.0130	119.2810
	GL-C1	40.19567*	6.17434	.000	18.5616	61.8297
	GL-C2	82.90567*	6.17434	.000	61.2716	104.5397
	GL-C3	51.17767*	6.17434	.000	29.5436	72.8117
	GL-C4	.00000	6.17434	1.000	-21.6340	21.6340
	GL-C6	.00000	6.17434	1.000	-21.6340	21.6340
	GL-C7	.00000	6.17434	1.000	-21.6340	21.6340
	GL-C0	-900.00000*	6.17434	.000	-921.6340	-878.3660
GL-C6	CURCUMIN	97.64700*	6.17434	.000	76.0130	119.2810
	GL-C1	40.19567*	6.17434	.000	18.5616	61.8297
	GL-C2	82.90567*	6.17434	.000	61.2716	104.5397
	GL-C3	51.17767*	6.17434	.000	29.5436	72.8117
	GL-C4	.00000	6.17434	1.000	-21.6340	21.6340
	GL-C5	.00000	6.17434	1.000	-21.6340	21.6340
	GL-C7	.00000	6.17434	1.000	-21.6340	21.6340
	GL-C0	-900.00000*	6.17434	.000	-921.6340	-878.3660
GL-C7	CURCUMIN	97.64700*	6.17434	.000	76.0130	119.2810
	GL-C1	40.19567*	6.17434	.000	18.5616	61.8297
	GL-C2	82.90567*	6.17434	.000	61.2716	104.5397
	GL-C3	51.17767*	6.17434	.000	29.5436	72.8117
	GL-C4	.00000	6.17434	1.000	-21.6340	21.6340
	GL-C5	.00000	6.17434	1.000	-21.6340	21.6340
	GL-C6	.00000	6.17434	1.000	-21.6340	21.6340
	GL-C0	-900.00000*	6.17434	.000	-921.6340	-878.3660
GL-C0	CURCUMIN	997.64700*	6.17434	.000	976.0130	1019.2810
	GL-C1	940.19567*	6.17434	.000	918.5616	961.8297
	GL-C2	982.90567*	6.17434	.000	961.2716	1004.5397
	GL-C3	951.17767*	6.17434	.000	929.5436	972.8117
	GL-C4	900.00000*	6.17434	.000	878.3660	921.6340
	GL-C5	900.00000*	6.17434	.000	878.3660	921.6340
	GL-C6	900.00000*	6.17434	.000	878.3660	921.6340
	GL-C7	900.00000*	6.17434	.000	878.3660	921.6340

*. The mean difference is significant at the 0.05 level.

ANOVA

IC50

	Sum of Squares	df	Mean Square	F	Sig.
Between Groups	2360437.078	8	295054.635	5.160E3	.000
Within Groups	1029.307	18	57.184		
Total	2361466.385	26			

Multiple Comparison IC_{50} of PMDC05

Tukey HSD

(I) Fractions	(J) Fractions	Mean Difference (I-J)	Std. Error	Sig.	95% Confidence Interval Lower Bound	Upper Bound
CURCUMIN	GL-C1	-98.78600*	2.70832	.000	-108.2756	-89.2964
	GL-C2	-20.09867*	2.70832	.000	-29.5882	-10.6091
	GL-C3	-40.00333*	2.70832	.000	-49.4929	-30.5138
	GL-C4	-98.78600*	2.70832	.000	-108.2756	-89.2964
	GL-C5	-98.78600*	2.70832	.000	-108.2756	-89.2964
	GL-C6	-98.78600*	2.70832	.000	-108.2756	-89.2964
	GL-C7	-98.78600*	2.70832	.000	-108.2756	-89.2964
	GL-C0	-998.78600*	2.70832	.000	-1008.2756	-989.2964
GL-C1	CURCUMIN	98.78600*	2.70832	.000	89.2964	108.2756
	GL-C2	78.68733*	2.70832	.000	69.1978	88.1769
	GL-C3	58.78267*	2.70832	.000	49.2931	68.2722
	GL-C4	.00000	2.70832	1.000	-9.4896	9.4896
	GL-C5	.00000	2.70832	1.000	-9.4896	9.4896
	GL-C6	.00000	2.70832	1.000	-9.4896	9.4896
	GL-C7	.00000	2.70832	1.000	-9.4896	9.4896
	GL-C0	-900.00000*	2.70832	.000	-909.4896	-890.5104
GL-C2	CURCUMIN	20.09867*	2.70832	.000	10.6091	29.5882
	GL-C1	-78.68733*	2.70832	.000	-88.1769	-69.1978
	GL-C3	-19.90467*	2.70832	.000	-29.3942	-10.4151
	GL-C4	-78.68733*	2.70832	.000	-88.1769	-69.1978
	GL-C5	-78.68733*	2.70832	.000	-88.1769	-69.1978
	GL-C6	-78.68733*	2.70832	.000	-88.1769	-69.1978
	GL-C7	-78.68733*	2.70832	.000	-88.1769	-69.1978
	GL-C0	-978.68733*	2.70832	.000	-988.1769	-969.1978
GL-C3	CURCUMIN	40.00333*	2.70832	.000	30.5138	49.4929
	GL-C1	-58.78267*	2.70832	.000	-68.2722	-49.2931
	GL-C2	19.90467*	2.70832	.000	10.4151	29.3942
	GL-C4	-58.78267*	2.70832	.000	-68.2722	-49.2931
	GL-C5	-58.78267*	2.70832	.000	-68.2722	-49.2931
	GL-C6	-58.78267*	2.70832	.000	-68.2722	-49.2931
	GL-C7	-58.78267*	2.70832	.000	-68.2722	-49.2931
	GL-C0	-958.78267*	2.70832	.000	-968.2722	-949.2931
GL-C4	CURCUMIN	98.78600*	2.70832	.000	89.2964	108.2756
	GL-C1	.00000	2.70832	1.000	-9.4896	9.4896
	GL-C2	78.68733*	2.70832	.000	69.1978	88.1769
	GL-C3	58.78267*	2.70832	.000	49.2931	68.2722
	GL-C5	.00000	2.70832	1.000	-9.4896	9.4896
	GL-C6	.00000	2.70832	1.000	-9.4896	9.4896
	GL-C7	.00000	2.70832	1.000	-9.4896	9.4896

	GL-C0	-900.00000*	2.70832	.000	-909.4896	-890.5104
GL-C5	CURCUMIN	98.78600*	2.70832	.000	89.2964	108.2756
	GL-C1	.00000	2.70832	1.000	-9.4896	9.4896
	GL-C2	78.68733*	2.70832	.000	69.1978	88.1769
	GL-C3	58.78267*	2.70832	.000	49.2931	68.2722
	GL-C4	.00000	2.70832	1.000	-9.4896	9.4896
	GL-C6	.00000	2.70832	1.000	-9.4896	9.4896
	GL-C7	.00000	2.70832	1.000	-9.4896	9.4896
	GL-C0	-900.00000*	2.70832	.000	-909.4896	-890.5104
GL-C6	CURCUMIN	98.78600*	2.70832	.000	89.2964	108.2756
	GL-C1	.00000	2.70832	1.000	-9.4896	9.4896
	GL-C2	78.68733*	2.70832	.000	69.1978	88.1769
	GL-C3	58.78267*	2.70832	.000	49.2931	68.2722
	GL-C4	.00000	2.70832	1.000	-9.4896	9.4896
	GL-C5	.00000	2.70832	1.000	-9.4896	9.4896
	GL-C7	.00000	2.70832	1.000	-9.4896	9.4896
	GL-C0	-900.00000*	2.70832	.000	-909.4896	-890.5104
GL-C7	CURCUMIN	98.78600*	2.70832	.000	89.2964	108.2756
	GL-C1	.00000	2.70832	1.000	-9.4896	9.4896
	GL-C2	78.68733*	2.70832	.000	69.1978	88.1769
	GL-C3	58.78267*	2.70832	.000	49.2931	68.2722
	GL-C4	.00000	2.70832	1.000	-9.4896	9.4896
	GL-C5	.00000	2.70832	1.000	-9.4896	9.4896
	GL-C6	.00000	2.70832	1.000	-9.4896	9.4896
	GL-C0	-900.00000*	2.70832	.000	-909.4896	-890.5104
GL-C0	CURCUMIN	998.78600*	2.70832	.000	989.2964	1008.2756
	GL-C1	900.00000*	2.70832	.000	890.5104	909.4896
	GL-C2	978.68733*	2.70832	.000	969.1978	988.1769
	GL-C3	958.78267*	2.70832	.000	949.2931	968.2722
	GL-C4	900.00000*	2.70832	.000	890.5104	909.4896
	GL-C5	900.00000*	2.70832	.000	890.5104	909.4896
	GL-C6	900.00000*	2.70832	.000	890.5104	909.4896
	GL-C7	900.00000*	2.70832	.000	890.5104	909.4896

*. The mean difference is significant at the 0.05 level.

ANOVA

IC50

	Sum of Squares	df	Mean Square	F	Sig.
Between Groups	2341365.284	8	292670.660	2.660E4	.000
Within Groups	198.045	18	11.002		
Total	2341563.328	26			

Multiple Comparison IC_{50} of PC-3

Tukey HSD

(I) Fractions	(J) Fractions	Mean Difference (I-J)	Std. Error	Sig.	95% Confidence Interval Lower Bound	Upper Bound
CURCUMIN	GL-C1	-95.71267*	1.55915	.000	-101.1757	-90.2496
	GL-C2	-23.44700*	1.55915	.000	-28.9101	-17.9839
	GL-C3	-95.71267*	1.55915	.000	-101.1757	-90.2496
	GL-C4	-95.71267*	1.55915	.000	-101.1757	-90.2496
	GL-C5	-95.71267*	1.55915	.000	-101.1757	-90.2496
	GL-C6	-95.71267*	1.55915	.000	-101.1757	-90.2496
	GL-C7	-95.71267*	1.55915	.000	-101.1757	-90.2496
	GL-C0	-995.71267*	1.55915	.000	-1001.1757	-990.2496
GL-C1	CURCUMIN	95.71267*	1.55915	.000	90.2496	101.1757
	GL-C2	72.26567*	1.55915	.000	66.8026	77.7287
	GL-C3	.00000	1.55915	1.000	-5.4631	5.4631
	GL-C4	.00000	1.55915	1.000	-5.4631	5.4631
	GL-C5	.00000	1.55915	1.000	-5.4631	5.4631
	GL-C6	.00000	1.55915	1.000	-5.4631	5.4631
	GL-C7	.00000	1.55915	1.000	-5.4631	5.4631
	GL-C0	-900.00000*	1.55915	.000	-905.4631	-894.5369
GL-C2	CURCUMIN	23.44700*	1.55915	.000	17.9839	28.9101
	GL-C1	-72.26567*	1.55915	.000	-77.7287	-66.8026
	GL-C3	-72.26567*	1.55915	.000	-77.7287	-66.8026
	GL-C4	-72.26567*	1.55915	.000	-77.7287	-66.8026
	GL-C5	-72.26567*	1.55915	.000	-77.7287	-66.8026
	GL-C6	-72.26567*	1.55915	.000	-77.7287	-66.8026
	GL-C7	-72.26567*	1.55915	.000	-77.7287	-66.8026
	GL-C0	-972.26567*	1.55915	.000	-977.7287	-966.8026
GL-C3	CURCUMIN	95.71267*	1.55915	.000	90.2496	101.1757
	GL-C1	.00000	1.55915	1.000	-5.4631	5.4631
	GL-C2	72.26567*	1.55915	.000	66.8026	77.7287
	GL-C4	.00000	1.55915	1.000	-5.4631	5.4631
	GL-C5	.00000	1.55915	1.000	-5.4631	5.4631
	GL-C6	.00000	1.55915	1.000	-5.4631	5.4631
	GL-C7	.00000	1.55915	1.000	-5.4631	5.4631
	GL-C0	-900.00000*	1.55915	.000	-905.4631	-894.5369
GL-C4	CURCUMIN	95.71267*	1.55915	.000	90.2496	101.1757
	GL-C1	.00000	1.55915	1.000	-5.4631	5.4631
	GL-C2	72.26567*	1.55915	.000	66.8026	77.7287
	GL-C3	.00000	1.55915	1.000	-5.4631	5.4631
	GL-C5	.00000	1.55915	1.000	-5.4631	5.4631
	GL-C6	.00000	1.55915	1.000	-5.4631	5.4631
	GL-C7	.00000	1.55915	1.000	-5.4631	5.4631

	GL-C0	-900.00000*	1.55915	.000	-905.4631	-894.5369
GL-C5	CURCUMIN	95.71267*	1.55915	.000	90.2496	101.1757
	GL-C1	.00000	1.55915	1.000	-5.4631	5.4631
	GL-C2	72.26567*	1.55915	.000	66.8026	77.7287
	GL-C3	.00000	1.55915	1.000	-5.4631	5.4631
	GL-C4	.00000	1.55915	1.000	-5.4631	5.4631
	GL-C6	.00000	1.55915	1.000	-5.4631	5.4631
	GL-C7	.00000	1.55915	1.000	-5.4631	5.4631
	GL-C0	-900.00000*	1.55915	.000	-905.4631	-894.5369
GL-C6	CURCUMIN	95.71267*	1.55915	.000	90.2496	101.1757
	GL-C1	.00000	1.55915	1.000	-5.4631	5.4631
	GL-C2	72.26567*	1.55915	.000	66.8026	77.7287
	GL-C3	.00000	1.55915	1.000	-5.4631	5.4631
	GL-C4	.00000	1.55915	1.000	-5.4631	5.4631
	GL-C5	.00000	1.55915	1.000	-5.4631	5.4631
	GL-C7	.00000	1.55915	1.000	-5.4631	5.4631
	GL-C0	-900.00000*	1.55915	.000	-905.4631	-894.5369
GL-C7	CURCUMIN	95.71267*	1.55915	.000	90.2496	101.1757
	GL-C1	.00000	1.55915	1.000	-5.4631	5.4631
	GL-C2	72.26567*	1.55915	.000	66.8026	77.7287
	GL-C3	.00000	1.55915	1.000	-5.4631	5.4631
	GL-C4	.00000	1.55915	1.000	-5.4631	5.4631
	GL-C5	.00000	1.55915	1.000	-5.4631	5.4631
	GL-C6	.00000	1.55915	1.000	-5.4631	5.4631
	GL-C0	-900.00000*	1.55915	.000	-905.4631	-894.5369
GL-C0	CURCUMIN	995.71267*	1.55915	.000	990.2496	1001.1757
	GL-C1	900.00000*	1.55915	.000	894.5369	905.4631
	GL-C2	972.26567*	1.55915	.000	966.8026	977.7287
	GL-C3	900.00000*	1.55915	.000	894.5369	905.4631
	GL-C4	900.00000*	1.55915	.000	894.5369	905.4631
	GL-C5	900.00000*	1.55915	.000	894.5369	905.4631
	GL-C6	900.00000*	1.55915	.000	894.5369	905.4631
	GL-C7	900.00000*	1.55915	.000	894.5369	905.4631

*. The mean difference is significant at the 0.05 level.

ANOVA

IC50

	Sum of Squares	df	Mean Square	F	Sig.
Between Groups	2294531.150	8	286816.394	7.866E4	.000
Within Groups	65.636	18	3.646		
Total	2294596.786	26			

Multiple Comparison IC_{50} of CHANG

IC50
Tukey HSD

(I) Fractions	(J) Fractions	Mean Difference (I-J)	Std. Error	Sig.	95% Confidence Interval	
					Lower Bound	Upper Bound
CURCUMIN	GL-C1	-91.90000*	15.56526	.000	-146.4385	-37.3615
	GL-C2	-68.46667*	15.56526	.008	-123.0052	-13.9281
	GL-C3	-58.90000*	15.56526	.029	-113.4385	-4.3615
	GL-C4	-91.90000*	15.56526	.000	-146.4385	-37.3615
	GL-C5	-91.90000*	15.56526	.000	-146.4385	-37.3615
	GL-C6	-91.90000*	15.56526	.000	-146.4385	-37.3615
	GL-C7	-91.90000*	15.56526	.000	-146.4385	-37.3615
	GL-C0	-991.90000*	15.56526	.000	-1046.4385	-937.3615
GL-C1	CURCUMIN	91.90000*	15.56526	.000	37.3615	146.4385
	GL-C2	23.43333	15.56526	.839	-31.1052	77.9719
	GL-C3	33.00000	15.56526	.491	-21.5385	87.5385
	GL-C4	.00000	15.56526	1.000	-54.5385	54.5385
	GL-C5	.00000	15.56526	1.000	-54.5385	54.5385
	GL-C6	.00000	15.56526	1.000	-54.5385	54.5385
	GL-C7	.00000	15.56526	1.000	-54.5385	54.5385
	GL-C0	-900.00000*	15.56526	.000	-954.5385	-845.4615
GL-C2	CURCUMIN	68.46667*	15.56526	.008	13.9281	123.0052
	GL-C1	-23.43333	15.56526	.839	-77.9719	31.1052
	GL-C3	9.56667	15.56526	.999	-44.9719	64.1052
	GL-C4	-23.43333	15.56526	.839	-77.9719	31.1052
	GL-C5	-23.43333	15.56526	.839	-77.9719	31.1052
	GL-C6	-23.43333	15.56526	.839	-77.9719	31.1052
	GL-C7	-23.43333	15.56526	.839	-77.9719	31.1052
	GL-C0	-923.43333*	15.56526	.000	-977.9719	-868.8948
GL-C3	CURCUMIN	58.90000*	15.56526	.029	4.3615	113.4385
	GL-C1	-33.00000	15.56526	.491	-87.5385	21.5385
	GL-C2	-9.56667	15.56526	.999	-64.1052	44.9719
	GL-C4	-33.00000	15.56526	.491	-87.5385	21.5385
	GL-C5	-33.00000	15.56526	.491	-87.5385	21.5385
	GL-C6	-33.00000	15.56526	.491	-87.5385	21.5385
	GL-C7	-33.00000	15.56526	.491	-87.5385	21.5385
	GL-C0	-933.00000*	15.56526	.000	-987.5385	-878.4615
GL-C4	CURCUMIN	91.90000*	15.56526	.000	37.3615	146.4385
	GL-C1	.00000	15.56526	1.000	-54.5385	54.5385
	GL-C2	23.43333	15.56526	.839	-31.1052	77.9719
	GL-C3	33.00000	15.56526	.491	-21.5385	87.5385
	GL-C5	.00000	15.56526	1.000	-54.5385	54.5385
	GL-C6	.00000	15.56526	1.000	-54.5385	54.5385

	GL-C7	.00000	15.56526	1.000	-54.5385	54.5385
	GL-C0	-900.00000*	15.56526	.000	-954.5385	-845.4615
GL-C5	CURCUMIN	91.90000*	15.56526	.000	37.3615	146.4385
	GL-C1	.00000	15.56526	1.000	-54.5385	54.5385
	GL-C2	23.43333	15.56526	.839	-31.1052	77.9719
	GL-C3	33.00000	15.56526	.491	-21.5385	87.5385
	GL-C4	.00000	15.56526	1.000	-54.5385	54.5385
	GL-C6	.00000	15.56526	1.000	-54.5385	54.5385
	GL-C7	.00000	15.56526	1.000	-54.5385	54.5385
	GL-C0	-900.00000*	15.56526	.000	-954.5385	-845.4615
GL-C6	CURCUMIN	91.90000*	15.56526	.000	37.3615	146.4385
	GL-C1	.00000	15.56526	1.000	-54.5385	54.5385
	GL-C2	23.43333	15.56526	.839	-31.1052	77.9719
	GL-C3	33.00000	15.56526	.491	-21.5385	87.5385
	GL-C4	.00000	15.56526	1.000	-54.5385	54.5385
	GL-C5	.00000	15.56526	1.000	-54.5385	54.5385
	GL-C7	.00000	15.56526	1.000	-54.5385	54.5385
	GL-C0	-900.00000*	15.56526	.000	-954.5385	-845.4615
GL-C7	CURCUMIN	91.90000*	15.56526	.000	37.3615	146.4385
	GL-C1	.00000	15.56526	1.000	-54.5385	54.5385
	GL-C2	23.43333	15.56526	.839	-31.1052	77.9719
	GL-C3	33.00000	15.56526	.491	-21.5385	87.5385
	GL-C4	.00000	15.56526	1.000	-54.5385	54.5385
	GL-C5	.00000	15.56526	1.000	-54.5385	54.5385
	GL-C6	.00000	15.56526	1.000	-54.5385	54.5385
	GL-C0	-900.00000*	15.56526	.000	-954.5385	-845.4615
GL-C0	CURCUMIN	991.90000*	15.56526	.000	937.3615	1046.4385
	GL-C1	900.00000*	15.56526	.000	845.4615	954.5385
	GL-C2	923.43333*	15.56526	.000	868.8948	977.9719
	GL-C3	933.00000*	15.56526	.000	878.4615	987.5385
	GL-C4	900.00000*	15.56526	.000	845.4615	954.5385
	GL-C5	900.00000*	15.56526	.000	845.4615	954.5385
	GL-C6	900.00000*	15.56526	.000	845.4615	954.5385
	GL-C7	900.00000*	15.56526	.000	845.4615	954.5385

*. The mean difference is significant at the 0.05 level.

ANOVA

IC50

	Sum of Squares	df	Mean Square	F	Sig.
Between Groups	2271916.934	8	283989.617	781.445	.000
Within Groups	6541.487	18	363.416		
Total	2278458.421	26			

Chang Fractions				
		IC50		
1	curcumin	8.118081	8.121028	8.110652
2	GL-C1	100	100	100
3	GL-C2	75.46035	78.81598	75.40956
4	GL-C3	100	100	100
5	GL-C4	100	100	100
6	GL-C5	100	00	100
7	GL-C6	100	100	100
8	GL-C7	100	100	100
9	GL-C0	866.7082	856.1179	865.9231

Jurkat fractions			
	IC50		
curcumin	2.784972	2.173655	2.099833
GL-C1	85.03188	50.43787	43.94329
GL-C2	18.0817	16.67518	16.52587
GL-C3	53.873	43.69481	48.89947
GL-C4	100	100	00
GL-C5	100	100	100
GL-C6	100	100	100
GL-C7	100	100	100
GL-C0	1000	1000	1000

PC3 fractions				
		IC50		
1	curcumin	6.321612	4.738353	1.802268
2	GL-C1	100	100	100
3	GL-C2	33.79533	24.60772	24.80007
4	GL-C3	100	100	100
5	GL-C4	100	100	100
6	GL-C5	100	100	100
7	GL-C6	100	100	100
8	GL-C7	100	100	100
9	GL-C0	1000	1000	1000

PMDCO5 -Sub Fraction

	IC50		
curcumin	1.319191	1.177418	1.145869
GL-C1	100	100	>100
GL-C2	24.06124	20.2495	19.62751
GL-C3	52.25591	37.06251	34.3328
GL-C4	100	100	100
GL-C5	100	100	100
GL-C6	100	100	100
GL-C7	100	100	100
GL-C0	1000	1000	1000
