# Supplementary material for: Cytotoxic activity of Ganoderma weberianum-sichuanese isolated from the Lower Volta River Basin of Ghana against human prostate carcinoma (PC-3), leukemic T cell (Jurkat), and plasmacytoid dendritic cell (pDC)-derived acute leukemia (PMDC05) cell lines
Source: PLoS One. 2025 Jul 11;20(7):e0327087. doi: 10.1371/journal.pone.0327087 (PMC12250544; doi:10.1371/journal.pone.0327087)
Supplement: S2 Table — (PDF) [file pone.0327087.s002.pdf]

Multiple Comparisons Chang Sub Fractions

IC50

Tukey HSD

(I) Sub-Fractions	(J) Sub-Fractions	Mean Difference (I-J)	Std. Error	Sig.	95% Confidence Interval	
					Lower Bound	Upper Bound
CURCUMIN	GL-C2-1	-92.13526*	.55120	.000	-94.0871	-90.1834
	GL-C2-2	-92.13526*	.55120	.000	-94.0871	-90.1834
	GL-C2-3	-92.13526*	.55120	.000	-94.0871	-90.1834
	GL-C2-4	-92.13526*	.55120	.000	-94.0871	-90.1834
	GL-C2-5	-92.13526*	.55120	.000	-94.0871	-90.1834
	GL-C2-6	-92.13526*	.55120	.000	-94.0871	-90.1834
	GL-C2-7	-92.13526*	.55120	.000	-94.0871	-90.1834
	GL-C2-8	-92.13526*	.55120	.000	-94.0871	-90.1834
	GL-C2-9	-92.13526*	.55120	.000	-94.0871	-90.1834
GL-C2-1 GL-	CURCUMIN	92.13526*	.55120	.000	90.1834	94.0871
	GL-C2-2	.00000	.55120	1.000	-1.9518	1.9518
	GL-C2-3	.00000	.55120	1.000	-1.9518	1.9518
	GL-C2-4	.00000	.55120	1.000	-1.9518	1.9518
	GL-C2-5	.00000	.55120	1.000	-1.9518	1.9518
	GL-C2-6	.00000	.55120	1.000	-1.9518	1.9518
	GL-C2-7	.00000	.55120	1.000	-1.9518	1.9518
	GL-C2-8	.00000	.55120	1.000	-1.9518	1.9518
	GL-C2-9	.00000	.55120	1.000	-1.9518	1.9518
GL-C2-2	CURCUMIN	92.13526*	.55120	.000	90.1834	94.0871
	GL-C2-1	.00000	.55120	1.000	-1.9518	1.9518
	GL-C2-3	.00000	.55120	1.000	-1.9518	1.9518
	GL-C2-4	.00000	.55120	1.000	-1.9518	1.9518
	GL-C2-5	.00000	.55120	1.000	-1.9518	1.9518
	GL-C2-6	.00000	.55120	1.000	-1.9518	1.9518
	GL-C2-7	.00000	.55120	1.000	-1.9518	1.9518
	GL-C2-8	.00000	.55120	1.000	-1.9518	1.9518
	GL-C2-9	.00000	.55120	1.000	-1.9518	1.9518
GL-C2-3	CURCUMIN	92.13526*	.55120	.000	90.1834	94.0871
	GL-C2-1	.00000	.55120	1.000	-1.9518	1.9518
	GLC2-2	.00000	.55120	1.000	-1.9518	1.9518
	GL-C2-4	.00000	.55120	1.000	-1.9518	1.9518

	GL-C2-5	.00000	.55120	1.000	-1.9518	1.9518
	GL-C2-6	.00000	.55120	1.000	-1.9518	1.9518
	GL-C2-7	.00000	.55120	1.000	-1.9518	1.9518
	GL-C2-8	.00000	.55120	1.000	-1.9518	1.9518
	GL-C2-9	.00000	.55120	1.000	-1.9518	1.9518
GL-C2-4	CURCUMIN	92.13526*	.55120	.000	90.1834	94.0871
	GL-C2-1	.00000	.55120	1.000	-1.9518	1.9518
	GL-C2-2	.00000	.55120	1.000	-1.9518	1.9518
	GL-C2-3	.00000	.55120	1.000	-1.9518	1.9518
	GL-C2-5	.00000	.55120	1.000	-1.9518	1.9518
	C2-6	.00000	.55120	1.000	-1.9518	1.9518
	C2-7	.00000	.55120	1.000	-1.9518	1.9518
	C2-8	.00000	.55120	1.000	-1.9518	1.9518
	C2-9	.00000	.55120	1.000	-1.9518	1.9518
GL-C2-5	CURCUMIN	92.13526*	.55120	.000	90.1834	94.0871
	GL-C2-1	.00000	.55120	1.000	-1.9518	1.9518
	GL-C2-2	.00000	.55120	1.000	-1.9518	1.9518
	GL-C2-3	.00000	.55120	1.000	-1.9518	1.9518
	GL-C2-4	.00000	.55120	1.000	-1.9518	1.9518
	GL-C2-6	.00000	.55120	1.000	-1.9518	1.9518
	GL-C2-7	.00000	.55120	1.000	-1.9518	1.9518
	GL-C2-8	.00000	.55120	1.000	-1.9518	1.9518
	GL-C2-9	.00000	.55120	1.000	-1.9518	1.9518
GL-C2-6	CURCUMIN	92.13526*	.55120	.000	90.1834	94.0871
	GL-C2-1	.00000	.55120	1.000	-1.9518	1.9518
	GL-C2-2	.00000	.55120	1.000	-1.9518	1.9518
	GL-C2-3	.00000	.55120	1.000	-1.9518	1.9518
	GL-C2-4	.00000	.55120	1.000	-1.9518	1.9518
	GL-C2-5	.00000	.55120	1.000	-1.9518	1.9518
	GL-C2-7	.00000	.55120	1.000	-1.9518	1.9518
	GL-C2-8	.00000	.55120	1.000	-1.9518	1.9518
	GL-C2-9	.00000	.55120	1.000	-1.9518	1.9518
GL-C2-7	CURCUMIN	92.13526*	.55120	.000	90.1834	94.0871
	GL-C2-1	.00000	.55120	1.000	-1.9518	1.9518
	GL-C2-2	.00000	.55120	1.000	-1.9518	1.9518
	GL-C2-3	.00000	.55120	1.000	-1.9518	1.9518

	GL-C2-4	.00000	.55120	1.000	-1.9518	1.9518
	GL-C2-5	.00000	.55120	1.000	-1.9518	1.9518
	GL-C2-6	.00000	.55120	1.000	-1.9518	1.9518
	GL-C2-8	.00000	.55120	1.000	-1.9518	1.9518
	GL-C2-9	.00000	.55120	1.000	-1.9518	1.9518
GL-C2-8	CURCUMIN	92.13526*	.55120	.000	90.1834	94.0871
	C2-1	.00000	.55120	1.000	-1.9518	1.9518
	C2-2	.00000	.55120	1.000	-1.9518	1.9518
	C2-3	.00000	.55120	1.000	-1.9518	1.9518
	C2-4	.00000	.55120	1.000	-1.9518	1.9518
	C2-5	.00000	.55120	1.000	-1.9518	1.9518
	C2-6	.00000	.55120	1.000	-1.9518	1.9518
	C2-7	.00000	.55120	1.000	-1.9518	1.9518
	C2-9	.00000	.55120	1.000	-1.9518	1.9518
GL-C2-9	CURCUMIN	92.13526*	.55120	.000	90.1834	94.0871
	GL-C2-1	.00000	.55120	1.000	-1.9518	1.9518
	GL-C2-2	.00000	.55120	1.000	-1.9518	1.9518
	GL-C2-3	.00000	.55120	1.000	-1.9518	1.9518
	GL-C2-4	.00000	.55120	1.000	-1.9518	1.9518
	GL-C2-5	.00000	.55120	1.000	-1.9518	1.9518
	GL-C2-6	.00000	.55120	1.000	-1.9518	1.9518
	GL-C2-7	.00000	.55120	1.000	-1.9518	1.9518
	GL-C2-8	.00000	.55120	1.000	-1.9518	1.9518

*. The mean difference is significant at the 0.05 level.

ANOVA

IC50

	Sum of Squares	df	Mean Square	F	Sig.
Between Groups	22920.045	9	2546.672	5.588E3	.000
Within Groups	9.115	20	.456		
Total	22929.159	29			

Multiple Comparisons JURKAT Sub fractions

IC50

Tukey HSD

(I) Sub-Fractions		Mean Difference (I-J)	Std. Error	Sig.	95% Confidence Interval	
					Lower Bound	Upper Bound
CURCUMIN	GL-C2-1	-96.22235*	1.50302	.000	-101.5447	-90.9000
	GL-C2-2	-96.22235*	1.50302	.000	-101.5447	-90.9000
	GL-C2-3	-96.22235*	1.50302	.000	-101.5447	-90.9000
	GL-C2-4	-45.30680*	1.50302	.000	-50.6291	-39.9844
	GL-C2-5	-52.01236*	1.50302	.000	-57.3347	-46.6900
	GL-C2-6	-96.22235*	1.50302	.000	-101.5447	-90.9000
	GL-C2-7	-96.22235*	1.50302	.000	-101.5447	-90.9000
	GL-C2-8	-96.22235*	1.50302	.000	-101.5447	-90.9000
	GL-C2-9	-96.22235*	1.50302	.000	-101.5447	-90.9000
GL-C2-1	CURCUMIN	96.22235*	1.50302	.000	90.9000	101.5447
	GL-C2-2	.00000	1.50302	1.000	-5.3224	5.3224
	GL-C2-3	.00000	1.50302	1.000	-5.3224	5.3224
	GL-C2-4	50.91555*	1.50302	.000	45.5932	56.2379
	GL-C2-5	44.20999*	1.50302	.000	38.8876	49.5323
	GL-C2-6	.00000	1.50302	1.000	-5.3224	5.3224
	GL-C2-7	.00000	1.50302	1.000	-5.3224	5.3224
	GL-C2-8	.00000	1.50302	1.000	-5.3224	5.3224
	GL-C2-9	.00000	1.50302	1.000	-5.3224	5.3224
GL-C2-2	CURCUMIN	96.22235*	1.50302	.000	90.9000	101.5447
	GL-C2-1	.00000	1.50302	1.000	-5.3224	5.3224
	GL-C2-3	.00000	1.50302	1.000	-5.3224	5.3224
	GL-C2-4	50.91555*	1.50302	.000	45.5932	56.2379
	GL-C2-5	44.20999*	1.50302	.000	38.8876	49.5323
	GL-C2-6	.00000	1.50302	1.000	-5.3224	5.3224
	GL-C2-7	.00000	1.50302	1.000	-5.3224	5.3224
	GL-C2-8	.00000	1.50302	1.000	-5.3224	5.3224
	GL-C2-9	.00000	1.50302	1.000	-5.3224	5.3224
GL-C2-3	CURCUMIN	96.22235*	1.50302	.000	90.9000	101.5447
	GL-C2-1	.00000	1.50302	1.000	-5.3224	5.3224
	GLC2-2	.00000	1.50302	1.000	-5.3224	5.3224
	GL-C2-4	50.91555*	1.50302	.000	45.5932	56.2379

	GL-C2-5	44.20999[*]	1.50302	.000	38.8876	49.5323
	GL-C2-6	.00000	1.50302	1.000	-5.3224	5.3224
	GL-C2-7	.00000	1.50302	1.000	-5.3224	5.3224
	GL-C2-8	.00000	1.50302	1.000	-5.3224	5.3224
	GL-C2-9	.00000	1.50302	1.000	-5.3224	5.3224
GL-C2-4	CURCUMIN	45.30680[*]	1.50302	.000	39.9844	50.6291
	GL-C2-1	-50.91555[*]	1.50302	.000	-56.2379	-45.5932
	GL-C2-2	-50.91555[*]	1.50302	.000	-56.2379	-45.5932
	GL-C2-3	-50.91555[*]	1.50302	.000	-56.2379	-45.5932
	GL-C2-5	-6.70556[*]	1.50302	.007	-12.0279	-1.3832
	C2-6	-50.91555[*]	1.50302	.000	-56.2379	-45.5932
	C2-7	-50.91555[*]	1.50302	.000	-56.2379	-45.5932
	C2-8	-50.91555[*]	1.50302	.000	-56.2379	-45.5932
	C2-9	-50.91555[*]	1.50302	.000	-56.2379	-45.5932
GL-C2-5	CURCUMIN	52.01236[*]	1.50302	.000	46.6900	57.3347
	GL-C2-1	-44.20999[*]	1.50302	.000	-49.5323	-38.8876
	GL-C2-2	-44.20999[*]	1.50302	.000	-49.5323	-38.8876
	GL-C2-3	-44.20999[*]	1.50302	.000	-49.5323	-38.8876
	GL-C2-4	6.70556[*]	1.50302	.007	1.3832	12.0279
	GL-C2-6	-44.20999[*]	1.50302	.000	-49.5323	-38.8876
	GL-C2-7	-44.20999[*]	1.50302	.000	-49.5323	-38.8876
	GL-C2-8	-44.20999[*]	1.50302	.000	-49.5323	-38.8876
	GL-C2-9	-44.20999[*]	1.50302	.000	-49.5323	-38.8876
GL-C2-6	CURCUMIN	96.22235[*]	1.50302	.000	90.9000	101.5447
	GL-C2-1	.00000	1.50302	1.000	-5.3224	5.3224
	GL-C2-2	.00000	1.50302	1.000	-5.3224	5.3224
	GL-C2-3	.00000	1.50302	1.000	-5.3224	5.3224
	GL-C2-4	50.91555[*]	1.50302	.000	45.5932	56.2379
	GL-C2-5	44.20999[*]	1.50302	.000	38.8876	49.5323
	GL-C2-7	.00000	1.50302	1.000	-5.3224	5.3224
	GL-C2-8	.00000	1.50302	1.000	-5.3224	5.3224
	GL-C2-9	.00000	1.50302	1.000	-5.3224	5.3224
GL-C2-7	CURCUMIN	96.22235[*]	1.50302	.000	90.9000	101.5447
	GL-C2-1	.00000	1.50302	1.000	-5.3224	5.3224
	GL-C2-2	.00000	1.50302	1.000	-5.3224	5.3224
	GL-C2-3	.00000	1.50302	1.000	-5.3224	5.3224

	GL-C2-4	50.91555*	1.50302	.000	45.5932	56.2379
	GL-C2-5	44.20999*	1.50302	.000	38.8876	49.5323
	GL-C2-6	.00000	1.50302	1.000	-5.3224	5.3224
	GL-C2-8	.00000	1.50302	1.000	-5.3224	5.3224
	GL-C2-9	.00000	1.50302	1.000	-5.3224	5.3224
GL-C2-8	CURCUMIN	96.22235*	1.50302	.000	90.9000	101.5447
	GL-C2-1	.00000	1.50302	1.000	-5.3224	5.3224
	GL-C2-2	.00000	1.50302	1.000	-5.3224	5.3224
	GL-C2-3	.00000	1.50302	1.000	-5.3224	5.3224
	GL-C2-4	50.91555*	1.50302	.000	45.5932	56.2379
	GL-C2-5	44.20999*	1.50302	.000	38.8876	49.5323
	GL-C2-6	.00000	1.50302	1.000	-5.3224	5.3224
	GL-C2-7	.00000	1.50302	1.000	-5.3224	5.3224
	GL-C2-9	.00000	1.50302	1.000	-5.3224	5.3224
GL-C2-9	CURCUMIN	96.22235*	1.50302	.000	90.9000	101.5447
	GL-C2-1	.00000	1.50302	1.000	-5.3224	5.3224
	GL-C2-2	.00000	1.50302	1.000	-5.3224	5.3224
	GL-C2-3	.00000	1.50302	1.000	-5.3224	5.3224
	GL-C2-4	50.91555*	1.50302	.000	45.5932	56.2379
	GL-C2-5	44.20999*	1.50302	.000	38.8876	49.5323
	GL-C2-6	.00000	1.50302	1.000	-5.3224	5.3224
	GL-C2-7	.00000	1.50302	1.000	-5.3224	5.3224
	GL-C2-8	.00000	1.50302	1.000	-5.3224	5.3224

*. The mean difference is significant at the 0.05 level.

ANOVA

IC50

	Sum of Squares	df	Mean Square	F	Sig.
Between Groups	30432.764	9	3381.418	997.881	.000
Within Groups	67.772	20	3.389		
Total	30500.536	29			

Multiple Comparisons PC-3 Sub fractions

IC50
Tukey HSD

(I) Sub-Fractions		Mean Difference (I-J)	Std. Error	Sig.	95% Confidence Interval	
					Lower Bound	Upper Bound
CURCUMIN	GL-C2-1	2.38677	3.38425	.999	-9.5972	14.3708
	GL-C2-2	-94.87274*	3.38425	.000	-106.8567	-82.8888
	GL-C2-3	-94.87274*	3.38425	.000	-106.8567	-82.8888
	GL-C2-4	-79.71722*	3.38425	.000	-91.7012	-67.7332
	GL-C2-5	-94.87274*	3.38425	.000	-106.8567	-82.8888
	GL-C2-6	-94.87274*	3.38425	.000	-106.8567	-82.8888
	GL-C2-7	-94.87274*	3.38425	.000	-106.8567	-82.8888
	GL-C2-8	-94.87274*	3.38425	.000	-106.8567	-82.8888
	GL-C2-9	-94.87274*	3.38425	.000	-106.8567	-82.8888
GL-C2-1	CURCUMIN	-2.38677	3.38425	.999	-14.3708	9.5972
	GL-C2-2	-97.25950*	3.38425	.000	-109.2435	-85.2755
	GL-C2-3	-97.25950*	3.38425	.000	-109.2435	-85.2755
	GL-C2-4	-82.10399*	3.38425	.000	-94.0880	-70.1200
	GL-C2-5	-97.25950*	3.38425	.000	-109.2435	-85.2755
	GL-C2-6	-97.25950*	3.38425	.000	-109.2435	-85.2755
	GL-C2-7	-97.25950*	3.38425	.000	-109.2435	-85.2755
	GL-C2-8	-97.25950*	3.38425	.000	-109.2435	-85.2755
	GL-C2-9	-97.25950*	3.38425	.000	-109.2435	-85.2755
GL-C2-2	CURCUMIN	94.87274*	3.38425	.000	82.8888	106.8567
	GL-C2-1	97.25950*	3.38425	.000	85.2755	109.2435
	GL-C2-3	.00000	3.38425	1.000	-11.9840	11.9840
	GL-C2-4	15.15552*	3.38425	.007	3.1715	27.1395
	GL-C2-5	.00000	3.38425	1.000	-11.9840	11.9840
	GL-C2-6	.00000	3.38425	1.000	-11.9840	11.9840
	GL-C2-7	.00000	3.38425	1.000	-11.9840	11.9840
	GL-C2-8	.00000	3.38425	1.000	-11.9840	11.9840
	GL-C2-9	.00000	3.38425	1.000	-11.9840	11.9840
GL-C2-3	CURCUMIN	94.87274*	3.38425	.000	82.8888	106.8567
	GL-C2-1	97.25950*	3.38425	.000	85.2755	109.2435
	GLC2-2	.00000	3.38425	1.000	-11.9840	11.9840
	GL-C2-4	15.15552*	3.38425	.007	3.1715	27.1395

	GL-C2-5	.00000	3.38425	1.000	-11.9840	11.9840
	GL-C2-6	.00000	3.38425	1.000	-11.9840	11.9840
	GL-C2-7	.00000	3.38425	1.000	-11.9840	11.9840
	GL-C2-8	.00000	3.38425	1.000	-11.9840	11.9840
	GL-C2-9	.00000	3.38425	1.000	-11.9840	11.9840
GL-C2-4	CURCUMIN	79.71722*	3.38425	.000	67.7332	91.7012
	GL-C2-1	82.10399*	3.38425	.000	70.1200	94.0880
	GL-C2-2	-15.15552*	3.38425	.007	-27.1395	-3.1715
	GL-C2-3	-15.15552*	3.38425	.007	-27.1395	-3.1715
	GL-C2-5	-15.15552*	3.38425	.007	-27.1395	-3.1715
	C2-6	-15.15552*	3.38425	.007	-27.1395	-3.1715
	C2-7	-15.15552*	3.38425	.007	-27.1395	-3.1715
	C2-8	-15.15552*	3.38425	.007	-27.1395	-3.1715
	C2-9	-15.15552*	3.38425	.007	-27.1395	-3.1715
GL-C2-5	CURCUMIN	94.87274*	3.38425	.000	82.8888	106.8567
	GL-C2-1	97.25950*	3.38425	.000	85.2755	109.2435
	GL-C2-2	.00000	3.38425	1.000	-11.9840	11.9840
	GL-C2-3	.00000	3.38425	1.000	-11.9840	11.9840
	GL-C2-4	15.15552*	3.38425	.007	3.1715	27.1395
	GL-C2-6	.00000	3.38425	1.000	-11.9840	11.9840
	GL-C2-7	.00000	3.38425	1.000	-11.9840	11.9840
	GL-C2-8	.00000	3.38425	1.000	-11.9840	11.9840
	GL-C2-9	.00000	3.38425	1.000	-11.9840	11.9840
GL-C2-6	CURCUMIN	94.87274*	3.38425	.000	82.8888	106.8567
	GL-C2-1	97.25950*	3.38425	.000	85.2755	109.2435
	GL-C2-2	.00000	3.38425	1.000	-11.9840	11.9840
	GL-C2-3	.00000	3.38425	1.000	-11.9840	11.9840
	GL-C2-4	15.15552*	3.38425	.007	3.1715	27.1395
	GL-C2-5	.00000	3.38425	1.000	-11.9840	11.9840
	GL-C2-7	.00000	3.38425	1.000	-11.9840	11.9840
	GL-C2-8	.00000	3.38425	1.000	-11.9840	11.9840
	GL-C2-9	.00000	3.38425	1.000	-11.9840	11.9840
GL-C2-7	CURCUMIN	94.87274*	3.38425	.000	82.8888	106.8567
	GL-C2-1	97.25950*	3.38425	.000	85.2755	109.2435
	GL-C2-2	.00000	3.38425	1.000	-11.9840	11.9840
	GL-C2-3	.00000	3.38425	1.000	-11.9840	11.9840

	GL-C2-4	15.15552*	3.38425	.007	3.1715	27.1395
	GL-C2-5	.00000	3.38425	1.000	-11.9840	11.9840
	GL-C2-6	.00000	3.38425	1.000	-11.9840	11.9840
	GL-C2-8	.00000	3.38425	1.000	-11.9840	11.9840
	GL-C2-9	.00000	3.38425	1.000	-11.9840	11.9840
GL-C2-8	CURCUMIN	94.87274*	3.38425	.000	82.8888	106.8567
	C2-1	97.25950*	3.38425	.000	85.2755	109.2435
	C2-2	.00000	3.38425	1.000	-11.9840	11.9840
	C2-3	.00000	3.38425	1.000	-11.9840	11.9840
	C2-4	15.15552*	3.38425	.007	3.1715	27.1395
	C2-5	.00000	3.38425	1.000	-11.9840	11.9840
	C2-6	.00000	3.38425	1.000	-11.9840	11.9840
	C2-7	.00000	3.38425	1.000	-11.9840	11.9840
	C2-9	.00000	3.38425	1.000	-11.9840	11.9840
GL-C2-9	CURCUMIN	94.87274*	3.38425	.000	82.8888	106.8567
	GL-C2-1	97.25950*	3.38425	.000	85.2755	109.2435
	GL-C2-2	.00000	3.38425	1.000	-11.9840	11.9840
	GL-C2-3	.00000	3.38425	1.000	-11.9840	11.9840
	GL-C2-4	15.15552*	3.38425	.007	3.1715	27.1395
	GL-C2-5	.00000	3.38425	1.000	-11.9840	11.9840
	GL-C2-6	.00000	3.38425	1.000	-11.9840	11.9840
	GL-C2-7	.00000	3.38425	1.000	-11.9840	11.9840
	GL-C2-8	.00000	3.38425	1.000	-11.9840	11.9840

*. The mean difference is significant at the 0.05 level.

ANOVA

IC50

	Sum of Squares	df	Mean Square	F	Sig.
Between Groups	43179.348	9	4797.705	279.266	.000
Within Groups	343.594	20	17.180		
Total	43522.942	29			

Multiple Comparisons PMDCO5 -Sub Fraction

IC50

Tukey HSD

(I) Sub-Fractions	(J) Sub-Fractions	Mean Difference (I-J)	Std. Error	Sig.	95% Confidence Interval	
					Lower Bound	Upper Bound
CURCUMIN	GL-C2-1	-96.69781*	9.81772	.000	-131.4634	-61.9322
	GL-C2-2	-96.69781*	9.81772	.000	-131.4634	-61.9322
	GL-C2-3	-77.80330*	9.81772	.000	-112.5689	-43.0377
	GL-C2-4	-20.98218	9.81772	.525	-55.7478	13.7834
	GL-C2-5	-13.51231	9.81772	.921	-48.2779	21.2533
	GL-C2-6	-56.60103*	9.81772	.000	-91.3666	-21.8354
	GL-C2-7	-96.73338*	9.81772	.000	-131.4990	-61.9678
	GL-C2-8	-96.69781*	9.81772	.000	-131.4634	-61.9322
	GL-C2-9	-58.40252*	9.81772	.000	-93.1681	-23.6369
GL-C2-1	CURCUMIN	96.69781*	9.81772	.000	61.9322	131.4634
	GL-C2-2	.00000	9.81772	1.000	-34.7656	34.7656
	GL-C2-3	18.89451	9.81772	.654	-15.8711	53.6601
	GL-C2-4	75.71563*	9.81772	.000	40.9500	110.4812
	GL-C2-5	83.18550*	9.81772	.000	48.4199	117.9511
	GL-C2-6	40.09678*	9.81772	.016	5.3312	74.8624
	GL-C2-7	-.03556	9.81772	1.000	-34.8012	34.7300
	GL-C2-8	.00000	9.81772	1.000	-34.7656	34.7656
	GL-C2-9	38.29530*	9.81772	.024	3.5297	73.0609
GL-C2-2	CURCUMIN	96.69781*	9.81772	.000	61.9322	131.4634
	GL-C2-1	.00000	9.81772	1.000	-34.7656	34.7656
	GL-C2-3	18.89451	9.81772	.654	-15.8711	53.6601
	GL-C2-4	75.71563*	9.81772	.000	40.9500	110.4812
	GL-C2-5	83.18550*	9.81772	.000	48.4199	117.9511
	GL-C2-6	40.09678*	9.81772	.016	5.3312	74.8624
	GL-C2-7	-.03556	9.81772	1.000	-34.8012	34.7300
	GL-C2-8	.00000	9.81772	1.000	-34.7656	34.7656
	GL-C2-9	38.29530*	9.81772	.024	3.5297	73.0609
GL-C2-3	CURCUMIN	77.80330*	9.81772	.000	43.0377	112.5689
	GL-C2-1	-18.89451	9.81772	.654	-53.6601	15.8711
	GLC2-2	-18.89451	9.81772	.654	-53.6601	15.8711
	GL-C2-4	56.82112*	9.81772	.000	22.0555	91.5867

	GL-C2-5	64.29099*	9.81772	.000	29.5254	99.0566
	GL-C2-6	21.20227	9.81772	.511	-13.5633	55.9679
	GL-C2-7	-18.93007	9.81772	.652	-53.6957	15.8355
	GL-C2-8	-18.89451	9.81772	.654	-53.6601	15.8711
	GL-C2-9	19.40079	9.81772	.623	-15.3648	54.1664
GL-C2-4	CURCUMIN	20.98218	9.81772	.525	-13.7834	55.7478
	GL-C2-1	-75.71563*	9.81772	.000	-110.4812	-40.9500
	GL-C2-2	-75.71563*	9.81772	.000	-110.4812	-40.9500
	GL-C2-3	-56.82112*	9.81772	.000	-91.5867	-22.0555
	GL-C2-5	7.46987	9.81772	.998	-27.2957	42.2355
	C2-6	-35.61885*	9.81772	.042	-70.3845	-.8532
	C2-7	-75.75119*	9.81772	.000	-110.5168	-40.9856
	C2-8	-75.71563*	9.81772	.000	-110.4812	-40.9500
	C2-9	-37.42033*	9.81772	.029	-72.1859	-2.6547
GL-C2-5	CURCUMIN	13.51231	9.81772	.921	-21.2533	48.2779
	GL-C2-1	-83.18550*	9.81772	.000	-117.9511	-48.4199
	GL-C2-2	-83.18550*	9.81772	.000	-117.9511	-48.4199
	GL-C2-3	-64.29099*	9.81772	.000	-99.0566	-29.5254
	GL-C2-4	-7.46987	9.81772	.998	-42.2355	27.2957
	GL-C2-6	-43.08872*	9.81772	.008	-77.8543	-8.3231
	GL-C2-7	-83.22107*	9.81772	.000	-117.9867	-48.4555
	GL-C2-8	-83.18550*	9.81772	.000	-117.9511	-48.4199
	GL-C2-9	-44.89021*	9.81772	.006	-79.6558	-10.1246
GL-C2-6	CURCUMIN	56.60103*	9.81772	.000	21.8354	91.3666
	GL-C2-1	-40.09678*	9.81772	.016	-74.8624	-5.3312
	GL-C2-2	-40.09678*	9.81772	.016	-74.8624	-5.3312
	GL-C2-3	-21.20227	9.81772	.511	-55.9679	13.5633
	GL-C2-4	35.61885*	9.81772	.042	.8532	70.3845
	GL-C2-5	43.08872*	9.81772	.008	8.3231	77.8543
	GL-C2-7	-40.13235*	9.81772	.016	-74.8979	-5.3667
	GL-C2-8	-40.09678*	9.81772	.016	-74.8624	-5.3312
	GL-C2-9	-1.80149	9.81772	1.000	-36.5671	32.9641
GL-C2-7	CURCUMIN	96.73338*	9.81772	.000	61.9678	131.4990
	GL-C2-1	.03556	9.81772	1.000	-34.7300	34.8012
	GL-C2-2	.03556	9.81772	1.000	-34.7300	34.8012
	GL-C2-3	18.93007	9.81772	.652	-15.8355	53.6957

	GL-C2-4	75.75119*	9.81772	.000	40.9856	110.5168
	GL-C2-5	83.22107*	9.81772	.000	48.4555	117.9867
	GL-C2-6	40.13235*	9.81772	.016	5.3667	74.8979
	GL-C2-8	.03556	9.81772	1.000	-34.7300	34.8012
	GL-C2-9	38.33086*	9.81772	.024	3.5653	73.0965
GL-C2-8	CURCUMIN	96.69781*	9.81772	.000	61.9322	131.4634
	C2-1	.00000	9.81772	1.000	-34.7656	34.7656
	C2-2	.00000	9.81772	1.000	-34.7656	34.7656
	C2-3	18.89451	9.81772	.654	-15.8711	53.6601
	C2-4	75.71563*	9.81772	.000	40.9500	110.4812
	C2-5	83.18550*	9.81772	.000	48.4199	117.9511
	C2-6	40.09678*	9.81772	.016	5.3312	74.8624
	C2-7	-.03556	9.81772	1.000	-34.8012	34.7300
	C2-9	38.29530*	9.81772	.024	3.5297	73.0609
GL-C2-9	CURCUMIN	58.40252*	9.81772	.000	23.6369	93.1681
	GL-C2-1	-38.29530*	9.81772	.024	-73.0609	-3.5297
	GL-C2-2	-38.29530*	9.81772	.024	-73.0609	-3.5297
	GL-C2-3	-19.40079	9.81772	.623	-54.1664	15.3648
	GL-C2-4	37.42033*	9.81772	.029	2.6547	72.1859
	GL-C2-5	44.89021*	9.81772	.006	10.1246	79.6558
	GL-C2-6	1.80149	9.81772	1.000	-32.9641	36.5671
	GL-C2-7	-38.33086*	9.81772	.024	-73.0965	-3.5653
	GL-C2-8	-38.29530*	9.81772	.024	-73.0609	-3.5297

*. The mean difference is significant at the 0.05 level.

ANOVA

IC50

	Sum of Squares	df	Mean Square	F	Sig.
Between Groups	38952.380	9	4328.042	29.935	.000
Within Groups	2891.628	20	144.581		
Total	41844.008	29			

Chang - Sub Fractions				
		IC50		
1	curcumin	10.29013	6.270768	7.033333
2	GL-C2-1	100	100	100
3	GL-C2-2	100	100	100
4	GL-C2-3	100	100	100
5	GL-C2-4	100	100	100
6	GL-C2-5	100	100	100
7	Gl-C2-6	100	100	100
8	GL-C2-7	100	100	100
9	GL-C2-8	100	100	100
10	GL-C2-9	100	100	100

JURKAT-Sub fractions			
	IC50		
curcumin	4.371673	3.536709	3.424582
GL-C2-1	100	100	100
GL-C2-2	100	100	100
GL-C2-3	100	100	100
GL-C2-4	47.38216	48.89077	50.98042
GL-C2-5	61.71071	54.84515	50.81417
Gl-C2-6	100	100	100
GL-C2-7	100	100	100
GL-C2-8	100	100	100
GL-C2-9	100	100	100

PC-3 Sub fractions				
		IC50		
1	curcumin	5.908185	4.208843	5.264757
2	GL-C2-1	1.736174	3.314207	3.171105
3	GL-C2-2	100	100	100
4	GL-C2-3	100	100	100
5	GL-C2-4	99.73939	75.42078	79.37327
6	GL-C2-5	100	100	100
7	Gl-C2-6	100	100	100
8	GL-C2-7	100	100	100
9	GL-C2-8	100	100	100
10	GL-C2-9	100	100	100

PMDCO5 Sub Fraction

	IC50		
curcumin	3.380264	3.25925	3.267048
GL-C2-1	100	100	100
GL-C2-2	100	100	100
GL-C2-3	86.72592	79.35243	77.23812
GL-C2-4	20.30554	32.95378	19.59379
GL-C2-5	23.3045	15.08271	12.05629
Gl-C2-6	76.93275	53.07049	49.70642
GL-C2-7	122.4952	91.6566	85.95492
GL-C2-8	100	100	100
GL-C2-9	92.75535	45.32412	47.03464
