# Supplementary material for: Cytotoxic activity of Ganoderma weberianum-sichuanese isolated from the Lower Volta River Basin of Ghana against human prostate carcinoma (PC-3), leukemic T cell (Jurkat), and plasmacytoid dendritic cell (pDC)-derived acute leukemia (PMDC05) cell lines
Source: PLoS One. 2025 Jul 11;20(7):e0327087. doi: 10.1371/journal.pone.0327087 (PMC12250544; doi:10.1371/journal.pone.0327087)
Supplement: Attachment — Submitted filename: Plos-Reviewers Comments.pdf [file pone.0327087.s003.pdf]

Review Comments to the Author

Reviewer 1

The article " Cytotoxic activity of Ganoderma weberianum-sichuanese isolated from Lower Volta River Basin of Ghana against human prostate carcinoma (PC-3), leukemic T cell (Jurkat) and plasmacytoid dendritic cell (pDC)-derived acute leukemia (PMDC05) cell lines" presents novel aspects on the evaluation of an extract of the fungus G. weberianum on the viability of cancer cells. However, I have some suggestions and comments to enrich the article.

1. Italicize the name Ganoderma weberianum- sichuanese in the title and in line 38.

Response: Italicized accordingly throughout the document (when not used as a common noun).

2. Line 92, "the fruiting body was surface disinfected with 70% alcohol".

Response: Thanks. Surfaced sterilized with 70% alcohol was replaced with surface disinfected with 70% alcohol as suggested

3. Specify what type of cells are the cell lines used in the experiments.

Response: The type of cells of the cell lines used are specified in Lines 33-36 and 41.

4. How were the subfractions made? What solvent was used?

Response: GL-C2 fraction was subjected to further silica column chromatography fractionation using varying mixtures of hexane, ethyl acetate, and methanol of increasing polarity to yield nine subfractions labeled GL-C2-C1 to C9. (See line 121-130)

5. Was TLC performed to qualitatively differentiate the different fractions?

Response: No, TLC was not performed in our current study because we were working with crude extracts. We primarily wanted to establish that the fractions would influence the viability of these cells in our current study. We selected the most active fractions (GL-C2-C1) because of its profound effect on Jurkat (IC50 = 17.09±0.86 µg/mL), PMDC05 (IC50 = 21.31±2.40 µg/mL), PC-3 (IC50 =27.73±5.25 µg/mL) and Chang liver cell (IC50 = 75.41±1.95 µg/mL) compared to the remaining fractions.

6. How was the calculation of the IC50 carried out, it is mentioned that through non-linear analysis, but what software was used for the calculation of this value?

Response: IC50 was calculated from the log dose-response curve using Excel.

7. In the figures it is mentioned that the mean is graphed, but are the error bars also presented?

Response: Thanks for bringing this to our attention. The correction has been made as follows 'Each point represents the mean value of the three replicates and the error bars represent the data distribution"

8. It is mentioned in line 191 that there is no significant difference, but the methodology does not describe what statistical analysis was performed in each assay.

Response: Thanks for the comment. This is a non-quantitative observation, so we have changed this accordingly to read "no observable cytotoxic effect".

9. The effect presented is on viability (analyzed by the MTT assay), not a cytotoxic effect.

Response: We have been following the nomenclature of the existing literature in this respect. For example, it is stated that, "Tetrazolium dye assays can also be used to measure cytotoxicity (loss of viable cells) or cytostatic activity (shift from proliferation to quiescence) of potential medicinal agents and toxic materials". We appreciate that this may be a matter of semantics and there are limitations when using this assay so we have addressed this in the discussions. Also, please see the following references:

Mosmann T. 1983. Rapid colorimetric assay for cellular growth and survival: Application to proliferation and cytotoxicity assays. Journal of Immunological Methods. 65(1-2):55-63. https://doi.org/10.1016/0022-1759(83)90303-4

Green LM, Reade JL, Ware CF. 1984. Rapid colormetric assay for cell viability: Application to the quantitation of cytotoxic and growth inhibitory lymphokines. Journal of Immunological Methods. 70(2):257-268. https://doi.org/10.1016/0022-1759(84)90190-x

10. Improve the images, they are not properly displayed in the document due to their low quality.

Response: Image quality has been improved for all Tables and Figures as suggested.

11. It remains to be identified what compounds the extract contains. The introduction talks about it, but its extracts lack any qualitative analysis of the presence of bioactive compounds. This is important to be able to identify the molecules responsible for the effect on the cell lines.

Response: We thank the reviewer for the comment regarding the identification of compounds present in our extracts and fractions and do agree this will ultimately be important. As we continue to scientifically explore the bioactivity of these extracts and fractions, we will identify compounds responsible for the biological activities observed in these studies. However, a rigerous molecular identification of the bioactive compounds was beyond the scope of this study so we ask for the reviewer's indulgence that this analysis will be carried out in a future study.

12. In table 2, place mL

Response: Thanks for the comment, ml was replaced with mL, the scientific notation for milliliters.

13. Check the mL units in the document. In some parts of the text it appears as ml.

Response: ml was replaced with mL throughout the document

14. Add perspectives in the conclusions, what is expected in the future with the study.

Response: Thank you, we have added additional text as suggested.

15. What was the particle size used to carry out the extraction?

Response: The particle size was 60 microns. We have added this to the methods section.

16. Specify how the extract was made. What extraction technique was used?

Response: As outlined in line 117 -120, mycelial biomass (300 g) was extracted with absolute ethanol, filtered, and the filtrates combined and dried by rotary evaporator.

17. I consider it more relevant to evaluate what compounds the fractions contain than to continue evaluating other cancer cells and animal models.

Response: Thanks for the comment. Evaluating the respective fractions on other cancer cells is a necessary step to determine if a full characterization would be justified. We agree that the purification and characterization of the bioactive compounds is important but was beyond the scope of this study and will be characterized in a follow-up study. We have articulated this is the discussions.

Reviewer #2:

1. Some background is missing from the abstract

Response: We have added additional text to the abstract to emphasize the medical importance of the Ganoderma genus.

2. The authors need more to highlight the originality of the work

Response: We have added this to the end of the abstract.

3. The authors don't explain if the extraction solvents were volatilized.

Response: Thank you, this was articulate in the methods section "dried by rotary evaporator". We have also emphasized this with respect to each fraction.

4. (2.3-0 36.80 μg/mL), review these data

Response: Thanks. Hyphen removed.

5. Indicate reference to determine the selectivity index

Response: Thanks. As indicated in line 169, extracts, fractions or subfractions with SI greater than 2 were considered to have good selectivity

6. Review the results because for the junkat cell line they report IC50 47.19 mg/mL and the graph does not show the decrease in viability for the GL-C1

Response: Thanks for drawing our attention to this mistake. The results have been reviewed to confirm GL-C1 did not decrease the viability of the jurkat cell line as shown in the graph.

7. Analysis is difficult due to the quality of the images

Response: The image resolution has been increased to improve quality.
