# Supplementary material for: Cytotoxic activity of Ganoderma weberianum-sichuanese isolated from the Lower Volta River Basin of Ghana against human prostate carcinoma (PC-3), leukemic T cell (Jurkat), and plasmacytoid dendritic cell (pDC)-derived acute leukemia (PMDC05) cell lines
Source: PLoS One. 2025 Jul 11;20(7):e0327087. doi: 10.1371/journal.pone.0327087 (PMC12250544; doi:10.1371/journal.pone.0327087)
Supplement: Attachment — Submitted filename: response to reviewers.pdf [file pone.0327087.s005.pdf]

Dear Reviewers,

The following comments have been addressed completely and without exception as indicated in red.

Thank you!

a) Specify the name and version of the software used for statistical analysis in the Methods section.

The software IBM SPSS statistics version 19 (IBM Inc., USA) was used for the statistical analysis. This has been added to the manuscript in the description of the statistical methods used (see below)

b) List all statistical tests performed and the threshold for significance (e.g., p-value).

The following paragraph has been added to the manuscript, under the heading "Statistical analysis" Statistical analyses were conducted using IBM SPSS Statistics version 19 (IBM Corp., USA). Given that the sample size was fewer than 30 per group, a one-way ANOVA was used, followed by Tukey's Honestly Significant Difference (HSD) post hoc test for multiple comparisons. A p-value of < 0.05 was considered statistically significant.

c) Verify and reconcile the IC_{50} values of curcumin reported in the text against those in Table 1.

IC50 values of curcumin in the cell lines in the texts have been corrected to agree with those in Table 1

d) Format all IC_{50} values as, for example, "5.13 ± 0.86 µg/mL" (with spaces around "±").

The IC50 with spaces have been changed accordingly in text and tables as recommended.

e) Correct the spelling of the cell line "PMDC05" in the main text (it currently appears as "PMDCo5" in tables).

PMDCo5 in the tables has been corrected to PMDC05 as it appears in the text.

f) Change all occurrences of "GL_C0" to "GL-C0" throughout the manuscript, including Figure 1.

“GL_C0” has been changed to “GL-C0” throughout the manuscript.

g) On line 199, specify the concentration range as “500–0 µg/mL.”

The range enter as 500-0 (Line 199) and 1000-500 (line 201) as recommended

h) Clarify whether the extract alone (without cells) was included as a background control.

Complete growth media, (without extract) cultured with cells was used as negative control. We have modified this to be clearer.

i) In the Results (line 343), add the appropriate citation for the statement made in that paragraph.

The following reference was added:

Jo, H. Y., Kim, Y., Park, H. W., Moon, H. E., Bae, S., Kim, J., Kim, D. G., & Paek, S. H. (2015). The Unreliability of MTT Assay in the Cytotoxic Test of Primary Cultured Glioblastoma Cells. *Experimental neurobiology*, *24*(3), 235–245. https://doi.org/10.5607/en.2015.24.3.235

Thank you!
