# Supplementary material for: Cytotoxic activity of Ganoderma weberianum-sichuanese isolated from the Lower Volta River Basin of Ghana against human prostate carcinoma (PC-3), leukemic T cell (Jurkat), and plasmacytoid dendritic cell (pDC)-derived acute leukemia (PMDC05) cell lines
Source: PLoS One. 2025 Jul 11;20(7):e0327087. doi: 10.1371/journal.pone.0327087 (PMC12250544; doi:10.1371/journal.pone.0327087)
Supplement: Attachment — Submitted filename: response to reviewers comments_2.docx [file pone.0327087.s004.docx]

Reviewer 1

1. If the authors have adequately addressed your comments raised in a previous round of review and you feel that this manuscript is now acceptable for publication, you may indicate that here to bypass the “Comments to the Author” section, enter your conflict of interest statement in the “Confidential to Editor” section, and submit your "Accept" recommendation.

All comments have been addressed

Response: Thanks.

2. Is the manuscript technically sound, and do the data support the conclusions?

The manuscript must describe a technically sound piece of scientific research with data that supports the conclusions. Experiments must have been conducted rigorously, with appropriate controls, replication, and sample sizes. The conclusions must be drawn appropriately based on the data presented.

Reviewer #1: Partly

Response: Thanks. The data presentation, discussion and conclusion as been improved as suggested

3. Has the statistical analysis been performed appropriately and rigorously?

Reviewer #1: I Don't Know

Thanks for the comment. The ANOVA statistical analysis comparing the EC_50_ between fractions has been performed. Statistical analysis of EC50 between sub-fractions was also performed and the data attached at supporting information.

4. Have the authors made all data underlying the findings in their manuscript fully available?

The [PLOS Data policy](http://www.plosone.org/static/policies.action#sharing) requires authors to make all data underlying the findings described in their manuscript fully available without restriction, with rare exception (please refer to the Data Availability Statement in the manuscript PDF file). The data should be provided as part of the manuscript or its supporting information, or deposited to a public repository. For example, in addition to summary statistics, the data points behind means, medians and variance measures should be available. If there are restrictions on publicly sharing data—e.g. participant privacy or use of data from a third party—those must be specified.

Reviewer #1: No

The raw data was uploaded to Dryad during the previous submission. The following is a link to the data or Dryad.

<https://datadryad.org/dataset/doi:10.5061/dryad.b5mkkwhmq>

5. Is the manuscript presented in an intelligible fashion and written in standard English?

PLOS ONE does not copyedit accepted manuscripts, so the language in submitted articles must be clear, correct, and unambiguous. Any typographical or grammatical errors should be corrected at revision, so please note any specific errors here.

Reviewer #1: Yes

Response: Thanks.

6. Review Comments to the Author

Please use the space provided to explain your answers to the questions above. You may also include additional comments for the author, including concerns about dual publication, research ethics, or publication ethics. (Please upload your review as an attachment if it exceeds 20,000 characters)

The comments above were results, however, due to the quality of the images in the previous review, comments regarding the results were not made. On this occasion, the observations made on the results are mentioned and it is recommended to address them, since the figures lack quality for publication.

Response: Thanks for this comment. The quality of all figures have been improved and the graphs have been plotted this time using Graph Pad.

Line 105: Correct “BLASTn”.

Response: Thanks for the comment. BLASTn has been appropriately and corrected to BLASTN

Line 135: Correct to understand what is going to be done in the fractions. “The cytotoxic activity test was carried out on the crude ethanolic extract (GL-CO1) and the seven fractions obtained”.

Response: Thanks for the comment. GC-CO1 has been corrected to GC-C0

Line 161: Place from highest to lowest concentration: "36.80-2.30 μg/mL".

Response. Thanks for the comment. Changed from 2.30-36.80 μg/mL to 36.80-2.30 μg/mL" to reflect highest to lowest.

Line 223: "Chang liver cell but a moderate cytotoxic effect on Jurkat with IC50 value 224 of 47.19±4.59 μg/mL and SI =2.12 (Figure 4A and Table 1)." This information does not correspond to what appears in Table 1.

Response. Thanks for the comment. The statement corrected to confirm the GL-C1 showed no cytotoxic effect against all the four cell lines as shown in Figure 4A and Table 1

Line 314: “This finding suggests that GL-C2-C1 may have a stronger anticancer activity against PC-3 compared to curcumin and could be useful in developing ganoderma mushroom-based products for treating human prostate carcinoma.” Was a statistical analysis performed to validate this assertion?

Response. Thanks for the comment. The p-value = 0.999 and therefore did not provide sufficient evidence to support the above statement. The statement has been modified accordingly.

This finding suggests that GL-C2-C1 may have a stronger anticancer activity against PC-3 similar compared to curcumin (p = 0.999) and could be useful like curcumin in developing ganoderma mushroom-based products for treating human prostate carcinoma.

Line 344. This could explain the results greater than 100% viability.

Thank you for your comment. We agree with this statement.

Why are two IC50 values for curcumin presented in each of the tables and an average is not made for the different experiments with their 3 independent experiments carried out?

A statistical analysis remains to be performed between the fractions to determine if they present a difference between the cytotoxic effects.

It remains to be mentioned whether the data is available.

Response: Thanks for the comment. See attached the statistical data showing the differences in the cytotoxic effects of the curcumin, extracts and fractions

The discussion needs to be improved, if the compounds present are unknown, try to address the explanation then under the extraction conditions with the different solvents and polarities used.

Response: Thanks for the comment

The discussion has been improved. Thank you for your comment.

In the previous review, the images could not be reviewed due to low quality, so in this review, pertinent comments are made to the figures:

Response: Thanks for the comment. Image quality has been improved for Figure 1-5.

Figure 1. Place GL-C0 instead of GL-ethanol extract.

Response: Thanks for the comment. GL-ethanol extract replaced with GL-C0 in Figure 1

Figure 3. A. Remove the comma ", PMDC05". Adjust the axes, start the X axis at 0, remove the -200 (A) and -20 (B). Check the error bars in Figure 3(B). They do not present the same format as (A).

Response: Thanks for the comment. The graph has been replotted using Graphpad

Figure 4. The information in Figure 3 is repeated here (GL-C01). Place the Figures in the same format. Same font size in the figure title. Check that the text of the axes does not overlap. The x axis in some graphs reaches up to 150 and in others up to 120 µg/mL. If the colors of the cell type lines are the same for all graphs, a single legend can be placed for all images. Figure 4 (C) Correct pDC to PMDC05.

Response: Thanks for the comment. Graph replotted and correction made in Figures 3 and 4

Figure 5. Same comment in Figure 5 regarding the format and that the text does not overlap on the axes. (A) Why is there a % viability of less than 0%? Is there any control? (H) For the PMDC05 cell line, 0 is presented at all concentrations, check if it is correct. If the colors of the cell type lines are the same for all graphs, a single legend can be placed for all images. (E) Check the error bar at the concentration of 100 g/mL for PC-3. It's correct? Since it presents a lot of dispersion in the result. (F) The error bar is not presented for PMDC05.

Response: Thanks for the comment. Graph replotted and corrections made

Table 1. Check GL-CO1, it should be GL-C0 according to the text.

Response: Thanks for the comment. GL-CO1 changed to GC-C0 in Table 1

7. PLOS authors have the option to publish the peer review history of their article ([what does this mean?](https://journals.plos.org/plosone/s/editorial-and-peer-review-process#loc-peer-review-history)). If published, this will include your full peer review and any attached files.


If you choose “no”, your identity will remain anonymous but your review may still be made public.


Do you want your identity to be public for this peer review? For information about this choice, including consent withdrawal, please see our [Privacy Policy](https://www.plos.org/privacy-policy).

Reviewer #1  No

Response: Thanks for the comment

Reviewer #2:

Cytotoxic activity of Ganoderma weberianum-sichuanese isolated from the Lower Volta River Basin of Ghana against human prostate carcinoma PC-3, leukemic T cell Jurkat, and plasmacytoid dendritic cell pDC-derived acute leukemia PMDC05 cell lines.

PLOS ONE

Comments to the Author

1. If the authors have adequately addressed your comments raised in a previous round of review and you feel that this manuscript is now acceptable for publication, you may indicate that here to bypass the “Comments to the Author” section, enter your conflict of interest statement in the “Confidential to Editor” section, and submit your "Accept" recommendation.

All comments have been addressed

Response: Thanks.

2. Is the manuscript technically sound, and do the data support the conclusions?

The manuscript must describe a technically sound piece of scientific research with data that supports the conclusions. Experiments must have been conducted rigorously, with appropriate controls, replication, and sample sizes. The conclusions must be drawn appropriately based on the data presented.

Reviewer #2: Yes

Response: Thanks.

3. Has the statistical analysis been performed appropriately and rigorously?

Reviewer #2: No

Thanks for the comment. The ANOVA statistical analysis comparing the EC_50_ between fractions has been performed. Statistical analysis of EC50 between sub-fractions was also performed and the data attached

4. Have the authors made all data underlying the findings in their manuscript fully available?

The [PLOS Data policy](http://www.plosone.org/static/policies.action#sharing) requires authors to make all data underlying the findings described in their manuscript fully available without restriction, with rare exception (please refer to the Data Availability Statement in the manuscript PDF file). The data should be provided as part of the manuscript or its supporting information, or deposited to a public repository. For example, in addition to summary statistics, the data points behind means, medians and variance measures should be available. If there are restrictions on publicly sharing data—e.g. participant privacy or use of data from a third party—those must be specified.

Reviewer #2: Yes

Response: Thanks for the comment

 5. Is the manuscript presented in an intelligible fashion and written in standard English?

PLOS ONE does not copyedit accepted manuscripts, so the language in submitted articles must be clear, correct, and unambiguous. Any typographical or grammatical errors should be corrected at revision, so please note any specific errors here.

Reviewer #2: Yes

 Response: Thanks for the comment

Reviewer #2:

Include if the same solvent ratios were used for the GL-C2 subfractions.
On line 162, include if the medium with the biomass was removed??? And if a control sample without cells was used.
Include in formula format the % to determine feasibility and SI
Include reference to compare with the Chang liver cell line if not all cancer lines are of liver origin

Response: Thanks for the comment. The formula has been stated in words this time in the manuscript

When the decrease in viability is less than 30%, it could be referred to as a decrease, not as toxicity, according to the ISO standard…

Response: Thanks for the comment. This is why we considered 50% decrease to represent cytotoxic effect in the study.

On line 228 specify concentration

Response: Thanks for the comments. The error has been addressed this time in the manuscript.

The PC3 labels are missing in figure 5

Response: Thanks for the comments. Figure 5 has been replotted and error addressed

In relation to the GL-C2-C8 subfraction in Figure 5, a viability of 0 is observed in all concentrations. Is this information correct?

Response: Thanks for the comments. This is true.

On line 332 discuss the results do not rewrite the same results

This has been addressed, thank you.

Include the entire statistical part in the methodology, include a reference for the description of moderate or low in terms of the decrease in viability.

This has been addressed, thank you.

7. PLOS authors have the option to publish the peer review history of their article ([what does this mean?](https://journals.plos.org/plosone/s/editorial-and-peer-review-process#loc-peer-review-history)). If published, this will include your full peer review and any attached files.


If you choose “no”, your identity will remain anonymous but your review may still be made public.


Do you want your identity to be public for this peer review? For information about this choice, including consent withdrawal, please see our [Privacy Policy](https://www.plos.org/privacy-policy).

Reviewer #2: No

Response: Thanks for the comment
